# Change in the Structural and Mechanical State of Heat-Resistant 15CrMoV5-10 Steel of TPP Steam Pipelines Under the Influence of Operational Factors

**DOI:** 10.3390/ma18143421

**Published:** 2025-07-21

**Authors:** Oleksandra Student, Halyna Krechkovska, Robert Pała, Ivan Tsybailo

**Affiliations:** 1 Department of Diagnostics of Materials Corrosion-Hydrogen Degradation, Karpenko Physico-Mechanical Institute of the NAS of Ukraine, 5 Naukova Str., 79060 Lviv, Ukraine; oleksandrastudent1@gmail.com (O.S.); tsybailo.14@gmail.com (I.T.); 2 Department of Materials Science and Engineering, Lviv Polytechnic National University, 12 S. Bandera Str., 79013 Lviv, Ukraine; 3 Faculty of Mechatronics and Mechanical Engineering, Kielce University of Technology, Av. 1000-An. of Polish State, 7, 25-314 Kielce, Poland; rpala@tu.kielce.pl

**Keywords:** heat-resistant steel, microstructure, mechanical properties, fractographic features, hydrogenation, fracture toughness

## Abstract

The operational efficiency of the main steam pipelines at thermal power plants is reduced due to several factors, including operating temperature, pressure, service life, and the frequency of process shutdowns, which contribute to the degradation of heat-resistant steels. The study aims to identify the features of changes in the sizes of grains and carbides along their boundaries, as well as mechanical properties (hardness, strength, plasticity and fracture toughness) along the wall thickness of both pipes in the initial state and after operation with block shutdowns. Preliminary electrolytic hydrogenation of specimens (before tensile tests in air) showed even more clearly the negative consequences of operational degradation of steel. The degradation of steel was also assessed using fracture toughness (*J*_IC_). The value of *J*_IC_ for operated steel with a smaller number of shutdowns decreased by 32–33%, whereas with a larger number of shutdowns, its decrease in the vicinity of the outer and inner surfaces of the pipe reached 65 and 61%, respectively. Fractographic signs of more intense degradation of steel after a greater number of shutdowns were manifested at the stage of spontaneous fracture of specimens by changing the mechanism from transgranular cleavage to intergranular, which indicated a decrease in the cohesive strength of grain boundaries.

## 1. Introduction

High-temperature operating conditions of power equipment create a particular danger in the event of through damage with leakage of coolant under high pressure. In particular, the occurrence of such damage in thick-walled pipe elements of the main steam pipeline system of thermal power plants (TPPs) threatens both personnel and the environment, and is also accompanied by significant financial losses.

Determining the residual life of elements of long-term equipment (as a necessary condition for preventing their breakdowns) largely depends on the correct assessment of the technical state of the metal at a specific moment of its operation. In general, engineering approaches have already been developed that make it possible to predict the service life of structural elements with crack-like defects in the presence of reliable information about their dimensions and metal properties [1,2]. However, in the case of thick-walled pipes, it is important to take into account another feature: the properties of the metal of even unused pipes are most often not constant across the thickness of their walls, due to the technological nuances of their manufacture. Moreover, without empirical data, it is simply impossible to predict a priori the patterns of their change in metal that has been used for a long time under the complex influence of numerous operational factors. After all, the long-term combined effect of temperature–force (the temperature of steam in pipelines reaches 570 °C, and the pressure is 24 MPa) and corrosion–hydrogenating factors contributes to the degradation of heat-resistant steels, changing their original microstructure [3,4,5,6], physical and mechanical properties [7,8] and the dominant mechanism of fracture [9,10,11,12].

Due to the increase in grain size, the precipitation and coagulation of carbides along their boundaries after long-term use of such steels, and the strength, plasticity, and crack-resistance characteristics deteriorate [13,14,15,16,17,18]. Micropores are formed in exploited steels even with fluctuations in temperature and pressure in the system within regulated limits. As their number is measured in gigacycles, they also contribute to the formation of pores and the weakening of the cross-section of steam pipelines [19].

Frequent shutdowns of the technological process due to the forced operation of the units in shunting mode further contribute to the development of damage in heat-resistant steels and the loss of their functionality [20]. At the beginning of the operation of steam pipelines, their influence could be neglected. However, over time, such thermal changes (especially during complete cooling of the pipeline system during forced shutdowns of units) contribute to intergranular cracking of degraded steels. The main reason for this is the formation of a significant number of large carbides along the grain boundaries. Due to the significant mismatch between carbides and the ferrite matrix in type, lattice size, elastic and thermal characteristics [21,22,23,24,25,26,27], defects are formed along the grain boundaries, weakening the cohesion between adjacent grains. Heating and cooling of thick-walled pipe systems during start-up and shutdown of units is accompanied by the occurrence of additional thermal stresses in the pipe sections. Summed up with stresses from the internal pressure of the coolant, they can sometimes even reach critical values [28,29,30].

It is also important to take into account the influence of hydrogen absorbed by steel during operation on its degradation, as well as the uneven distribution of hydrogen across the pipe cross-section, which depends on both temperature and stress. Under the influence of hydrogen, the diffusion of alloying elements is accelerated [31] and, consequently, microstructural transformations in the metal are intensified with the appearance of pores [32], their merging, and the formation of intergranular cracks in the elements of steam pipelines.

Consequently, the comparatively fast stage of degradation associated with the fracture propagation to the entire cross-section of the structural element is preceded by a significantly slower stage, which is not yet associated with the formation of defects and cracks. It is manifested by a change in the structure of the metal at the microscopic level and, as a consequence, a gradual deterioration in its mechanical properties. At the same time, although at the macroscopic level the structural element still retains its integrity, steel that has been in use for a long time loses the properties that ensured its functionality at the beginning of its use. In particular, the creep rate of the steels in use increases [33], and strength and plasticity characteristics [8], as well as impact toughness and crack-growth resistance [12,28,34,35,36,37], decrease. In addition, it is irresponsible not to take into account the negative impact of hydrogen on the properties of long-exploited steels. After all, its concentration in exploited steel is higher than in unexploited steel [38,39]. Moreover, it is unevenly distributed across the cross-section of elements. It is known that its local content near crack tips, as well as near real fractures of steam pipelines, significantly exceeds its integral content [40].

Based on the analysis of factors influencing the degradation of heat-resistant steels during their long-term operation on the main steam pipelines of TPPs, it was recognized as important to analyze the features of changes in their microstructure and mechanical properties at different levels along the pipe wall thickness. In this case, special attention was paid to such factors of negative impact on the properties of heat-resistant steel as its hydrogenation and multiple start-up and shutdown of power units. It was believed that taking into account the gradient of steel properties across the wall thickness of operated pipes would make it possible to more accurately determine both the critical length of cracks and the residual life of steam pipeline elements after their specific operation under appropriate conditions.

The work aims to establish the patterns of change in the microstructure, and failure mechanisms and mechanical properties (strength characteristics, plasticity and fracture toughness) at different levels along the wall thickness of pipes (near their inner and outer surfaces and in the center of the cross section) made of 15CrMoV5-10 steel. This will be carried out in the initial state (reserve pipe) and after the same period of operation (1.9 × 10^5^ h) in pipes with a different number of block shutdowns (501 and 576) when testing specimens without and after electrolytic hydrogenation (to visualize signs of the negative impact of high-temperature degradation on mechanical properties).

## 2. Materials and Methods

Three variants of thick-walled pipes (outer diameter and wall thickness of 325 and 60 mm, respectively) made of low-alloy heat-resistant steel DIN 15CrMoV5-10 (corresponding to 15Kh1M1F steel according to Ukrainian Standard [41]) were compared. The chemical composition of this steel is wt.%: 0.16 C, 0.30 Si, 0.9 Mn, 1.39 Cr, 0.017 S, 0.0021 P, 0.2 Ni, 0.29 V, 0.97 Mo, and Fe for the rest. These pipes have undergone factory heat treatment (quenching in oil from 970 to 1000 °C with high tempering at 730–760 °C for 10 h). The first pipe is made of steel in the initial state (reserve pipe (marked No. 1)). The pipes of the second and third variants (marked No. 2 and No. 3, respectively) were subjected to long-term operation for about 1.9 × 10^5^ h on vertical sections of main steam pipelines of TPP at temperatures of up to 545 °C and a steam pressure of up to 24 MPa. Both operating pipes were subject to varying numbers of unit shutdowns with full or partial cooling by the steam pipeline systems. In particular, during the operation of steel on pipe No. 2, a smaller number of forced technical shutdowns were recorded, 502 times, than during operation on pipe No. 3—576 times.

Hardness *HB*, tensile mechanical properties (ultimate strength σ_UTS_ and yield strength σ_YS_, elongation *El* and reduction in area *RA*), and fracture toughness *J*_IC_ were used to certify the steel from each pipe. The NOVOTEST hardness tester (Novomoskovsk, Ukraine) was used to measure hardness. The data from at least 10 measurements at each level of the pipe wall thickness were averaged. The data scatter band did not exceed 5%.

**Figure 1:**
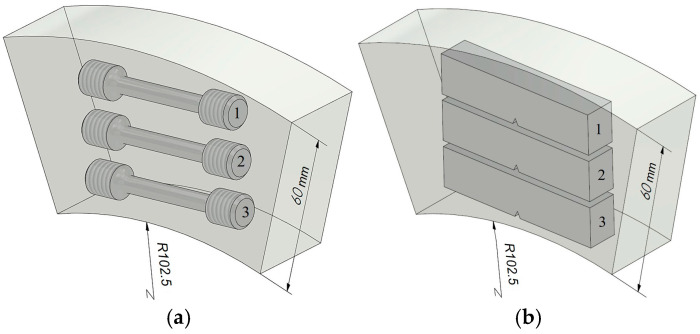
Schematic diagram of the arrangement of tangential specimens at different levels along the pipe wall thickness, intended for tensile testing (**a**) and fracture toughness (**b**). Numbers 1, 2 and 3 indicate the location of the specimens in the cross-section of the pipes, respectively, near the outer surface, in the center of the section and near their inner surface.

The mechanical properties of steel from three types of pipes were determined on transverse (tangentially oriented) specimens cut at different levels along the wall thickness of each pipe (near their outer and inner surfaces, as well as in the center of their cross-section, Figure 1). In this case, the axis of the specimens was located at a distance of 10, 30 and 55 mm from the outer surface of the pipes (with an accuracy of ±1 mm). To determine the mechanical properties under tension, cylindrical specimens with a diameter of 5 mm and a working part length of 25 mm were used. All specimens were tested in air at about 20 °C. Before the tensile test, some of them were electrolytically hydrogenated for 15 min in a 5% H_2_SO_4_ solution (pH 0) with the addition of 2 g/L thiourea at a current density of 50 mA/cm^2^. In this way, the consequences of the possible harmful effects of hydrogen, additionally absorbed by the metal during operation, were simulated. Tensile tests were carried out on an UME-10T testing machine in accordance with ISO 6892-1:2019 [42,43,44]. The deformation rate of the specimens was 10^–3^ s^–1^. Before testing, all specimens were polished, to remove traces of mechanical surface treatment, using polishing compounds of different grain sizes. The obtained characteristics were averaged based on the results of at least three tests, and the data scatter did not exceed 3–5%.

The fracture toughness of the studied steel variants was determined using the *J*-integral method, observing the requirements of the regulatory document [45,46]. Beam specimens measuring 10 × 15 × 75 mm with a one-sided fatigue crack from the notch tip with a total length of 7.5 mm were used. The fatigue-crack growth rate at the final stage of its formation (the last 1.5 mm of its length) was 5 × 10^–9^ m/cycle. The specimens were loaded according to the three-point bending scheme on a Zwick-100 testing machine with an electromechanical drive, equipped with an automated test control and data recording system from the Zwick Company (ZwickRoell, Ulm, Germany). To achieve the goal of the work (investigation of fracture-toughness change trends across the wall thickness of pipes), it was necessary to use specimens cut from different levels in the pipe sections, which limited their dimensions. As a result, the loading diagrams of the specimens used during fracture-toughness testing were nonlinear, which limited the applicability of linear fracture mechanics approaches to the analyzed steel. Therefore, the J-integral method was used, and its critical values, *J*_IC_, were determined. During the tests, the change in load and compliance of the specimen was monitored [47]. The dependences of the current values of the J-integral on the corresponding crack increment Δa (*J*_R_-curves) were obtained by the method of multiple partial unloading of specimens during their loading. Based on the *J*_R_-curves for each of the steel variants, the *J*_IC_ levels were determined, corresponding to the start of crack growth from the tips of the initial fatigue cracks under active loading of the specimens. As a feature of long-term exploited steel, it was noted that, after the beginning of subcritical growth of cracks in the specimens, their uncontrolled propagation began almost immediately. Therefore, the fracture-toughness values of both variants of the operated steel were determined using their loading diagram at the moment of uncontrolled crack propagation.

Metallographic studies of the microstructure of steels and fractographic features of their fracture surfaces were carried out using SEM EVO 40XVP (ZEISS, Oberkochen, Germany) and JSM-7100F (SEM, JEOL, Zaventem, Belgium).

## 3. Results and Discursion

**Figure 2:**
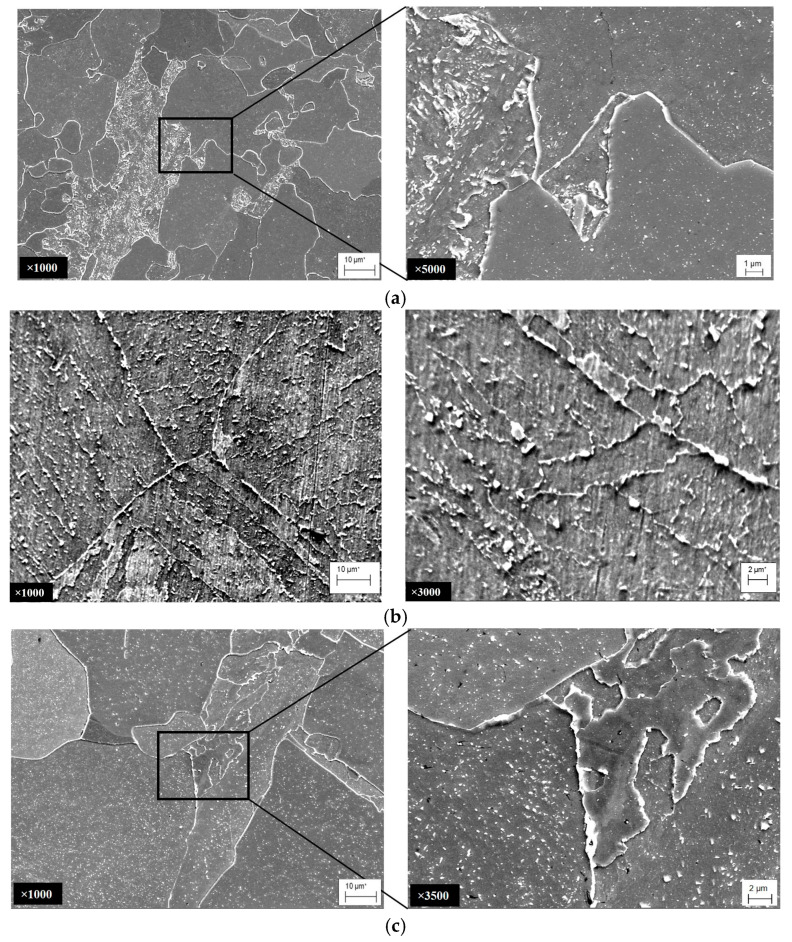
The microstructure of steel in the initial state (**a**) and after the long-term operation (approx. 1.9 × 10^5^ h) in main steam pipelines of TPP (**b**,**c**) after 502 (**b**) and 576 (**c**) unit shutdowns.

*Microstructural features of steel degradation.* A ferrite–bainitic microstructure with a fairly uniform distribution of ferrite and bainite grains across the pipe wall thickness prevailed in the unexploited pipe (No. 1), which was made of 15CrMoV5-10 steel (Figure 2a). Small carbides were observed inside the metastable bainite grains, which were precipitated during high-temperature tempering of the steel at the final stage of heat treatment of the pipe at the manufacturing plant. In the microstructure of steel that had been operating for a long time under the influence of high temperatures and stresses, clear signs of the disappearance of the boundaries between adjacent packets of high-temperature bainite were found (Figure 2b,c). Thermodynamically more stable secondary carbides enriched with Mo and Cr were precipitated. At the same time, carbon and alloying elements were redistributed to grain boundaries as thermodynamically more favorable locations for carbides. As a result, the content of alloying elements in the ferrite matrix decreased, and complex-alloyed chromium and molybdenum carbides were precipitated and coagulated along the grain boundaries. Similar changes in the microstructure as a result of operational degradation of heat-resistant steels are noted by other researchers [48,49,50].

**Figure 3:**
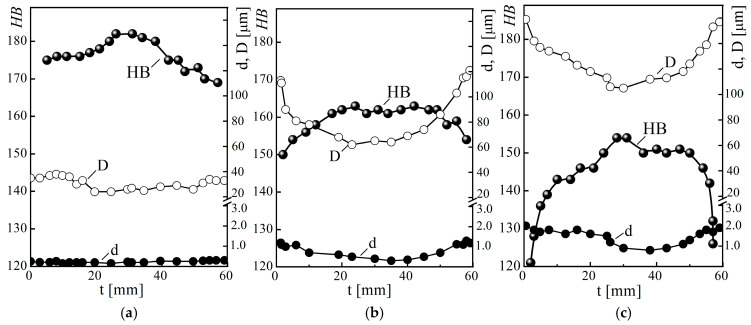
Regularities of change in the hardness HB, average grain size, *D*, and carbides, *d*, across the wall thickness of pipes *t* (starting from their outer surface), measured in the diametrical section of pipes made of 15CrMoV5-10 steel in the initial state ((**a**) No. 1) and after the same time of operation (approximately 1.9 × 10^5^ h) on the main steam pipeline of the TPP, but with a different number of unit shutdowns: 502 ((**b**) No. 2) and 576 ((**c**) No. 3).

It was also found that after long-term operation, both the grain size *D* and the gradient of its change along the pipe wall thickness *t* increased in the steel structure (Figure 3). Moreover, the negative impact of steel degradation on this structural indicator increased with the number of unit shutdowns during its operation. In particular, near the outer surface of pipes No. 2 and No. 3, the grain size increased by almost 3.2 and 4.5 times, relative to steel in the initial state (pipe No. 1), and near their inner surface, the difference between them increased even more (by 3.3 and 5.1 times, respectively), whereas in the center of the pipe cross-section, the increase in D (relative to its value for steel in the initial state) was noticeably smaller (only 2.2 and 3.7 times for steel from pipes No. 2 and No. 3, respectively).

**Figure 4:**
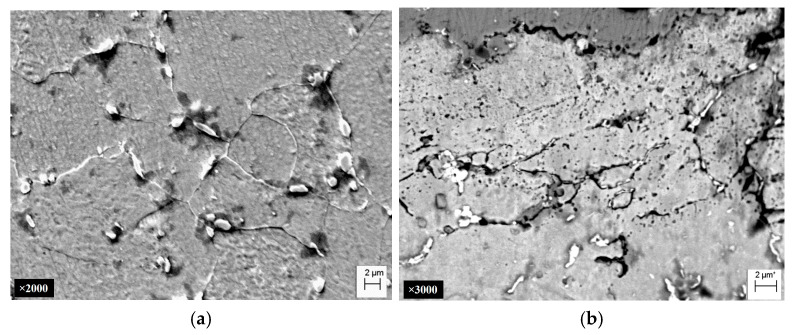
The arrangement of carbides along grain boundaries (**a**) and signs of intergranular oxidation (**b**) in the microstructure of 15CrMoV5-10 steel directly under the outer surface of pipe No. 3, which operated for about 1.9 × 10^5^ h on the main steam pipeline of a thermal power plant with a large number of unit shutdowns.

In general, the most noticeable changes in grain size occurred in the near-surface layers of pipes up to 10–15 mm thick (Figure 3). Overall, it was in these layers of the pipe cross-section that the most favorable conditions for steel degradation were created, due to the combined effect of steam pressure in the pipes and residual thermal stresses caused by block shutdowns [28]. In addition, the ferrite grain boundaries directly under the outer surface of the operated pipes were practically not visible (Figure 4a). Their location could be determined only due to the presence of chains of large carbides (up to 1.5–2 μm), which were precipitated and coagulated along the grain boundaries during operation. Studies of the structure near the outer surface of pipe No. 3 revealed fairly deep (from 20 to 70 μm) microcracks along the ferrite grain boundaries (Figure 4b). Under the combined effect of metal hydrogenation and local stresses arising during process shutdowns, carbides separated from the ferrite matrix and formed voids (due to the discrepancy between their thermal expansion coefficient, elastic modulus and lattice parameter, and the ferrite matrix). As a result of high-temperature creep during pipe operation, these voids merged, forming intergranular microcracks. Their surfaces were intensively oxidized, due to the almost unimpeded access of oxygen from the outside, and the products of high-temperature oxidation filled the space between the edges of these cracks. Since the volume of oxides is greater than the volume of the matrix, they additionally wedged these cracks and contributed to their further propagation during the cooling of the pipes during unit shutdowns.

Considering the key role of carbide precipitation along grain boundaries in the initiation of cracks from the outer surface of steam pipelines, it is also analyzed how their sizes change across the thickness of the pipe wall after the combined effect on the metal of both the operating time and the number of unit shutdowns (Figure 3). In the structure of steel in the initial state (pipe No. 1), only an insignificant amount of relatively small (up to 0.3 μm) carbides were found along the grain boundaries (Figure 2a). Moreover, their sizes remained virtually unchanged along the pipe wall thickness (Figure 3a). Overall, in this version of steel, carbon is mainly either in a solid solution or in small particles of cementite that were formed during the heat treatment of the pipe at the manufacturing plant. As a result of the operation of the steel, carbides were precipitated and coagulated along the grain boundaries as thermodynamically favorable locations for this (Figure 2b,c), and their sizes increased (Figure 3b,c). It was noted that the maximum growth of carbide sizes occurred near both surfaces of pipes No. 2 and No. 3, while in the center of their cross-sections the increase in carbide size was less noticeable. It was confirmed that after the operation, the trends in the size change of both carbides and grains in the cross-section of pipes are qualitatively similar. Moreover, the obtained trends in their change over the pipe wall thickness are consistent with the trend in stress change in the pipe wall due to block shutdowns. On this basis, it was assumed that thermal stresses caused by block shutdowns can also intensify the growth of these important components of the steel microstructure.

The described transformation of the steel structure during operation on steam pipelines (increase in the sizes of grains and carbides along their boundaries, decohesion of carbides from the matrix, and occurrence of pores at the grain boundaries) will affect its properties. The hardness HB for steel from operated pipes decreased (Figure 3b,c) relative to the hardness of steel in the initial state (Figure 3a), and the trends of its change along the pipe wall thickness were the opposite, relative to those observed for the sizes of grains and carbides (Figure 3). For all pipes, the maximum decrease in hardness HB was observed in the vicinity of both their surfaces (precisely where the sizes of both grains and carbides were the largest). Thus, near the outer and inner surfaces of operated pipe No. 2, the steel hardness decreased by 14 and 9%, and for pipe No. 3 by 31 and 25%, respectively, relative to the hardness of non-operated pipe No. 1. At the same time, in the center of the wall of pipe No. 2, the steel hardness decreased by 11%, and in pipe No. 3 by 18%. As a result, the hardness of the steel in the initial state, measured at a distance of 5 mm from the outer surface of the pipe, was 178 HB, while in the operated steel No. 2 (with a smaller number of shutdowns) it decreased to 156 HB, and in steel No. 3 (with a greater number of them) to 140 HB, respectively. As a result, almost one quarter of the cross-section of pipe No. 3 no longer met the requirements for heat-resistant steels in terms of hardness (145 HB). Consequently, despite the almost identical service life of pipes No. 2 and No. 3 on the steam pipeline, the negative impact of the increase in the number of unit shutdowns on the hardness of the steel was manifested at all levels of assessment by the wall thickness of the pipes, but it became most noticeable near both of their surfaces.

Thus, the degradation of 15CrMoV5-10 steel under the influence of operational factors manifested itself in the transformation of the microstructure, with an increase in the size of ferrite grains and the precipitation and coagulation of carbides along their boundaries. An increase in the number of block shutdowns (as initiators of thermal stresses, maximum near both surfaces of thick-walled pipes [28]) intensifies the growth of both microstructural components. As a result, firstly, the sizes of grains and carbides changed most strongly during operation near the side surfaces of pipes. Secondly, the revealed trends in the change in the sizes of microstructural components are opposite to the trend in the change in steel hardness. And thirdly, such changes caused the decarburization of high-tempered bainite, the disappearance of the boundaries between its neighboring packets, and the formation of a virtually ferrite–carbide structure in the operated steels.

**Figure 5:**
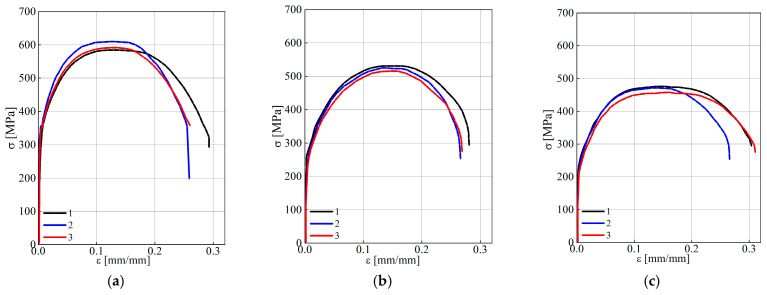
Typical loading diagrams of tangential specimens cut near the outer (1) and inner (3) surfaces and in the center of the cross-section (2) of pipes made of 15CrMoV5-10 steel in the initial state ((**a**) No. 1) and after operation, for approximately 1.9 × 10^5^ h on steam pipelines, accompanied by 502 ((**b**) No. 2) and 576 ((**c**) No. 3) process shutdowns.

*Trends in the change of mechanical properties of steel by the wall thickness of steam pipeline pipes.* In calculations of structural elements for strength, as a rule, the strength characteristics of materials obtained during their tensile testing are used, whereas the plasticity characteristics characterize the potential ability of the operated metal to plastically deform until reaching a critical state. Therefore, the analysis of the trends in the change of these characteristics by the wall thickness of steam pipeline pipes in the initial state and after operation with a different number of process shutdowns is important for ensuring the correctness of current calculations of steam pipeline elements for strength. Typical loading curves of the specimens of the analyzed steel variants, obtained during tensile tests in air, are shown in Figure 5, and their average characteristics, obtained during tensile tests in air, are given in Table 1. Analysis of the presented data showed that in the initial state of the steel (pipe No. 1), there is already an obvious tendency towards an increase in strength characteristics and a decrease in plasticity characteristics across the thickness of the pipe wall in the direction from its outer to the inner surface. In particular, the yield strength σ_YS_ of steel near the inner surface of the pipe exceeded its value near the outer surface by 11%, while its ultimate strength σ_UTS_ showed only a barely noticeable tendency for such an increase. At the same time, the elongation value of steel near the inner surface of the pipe (relative to its inherent value near the outer surface) decreased by 11%, and the RA values remained virtually unchanged across the thickness of the pipe wall.

**Figure 6:**
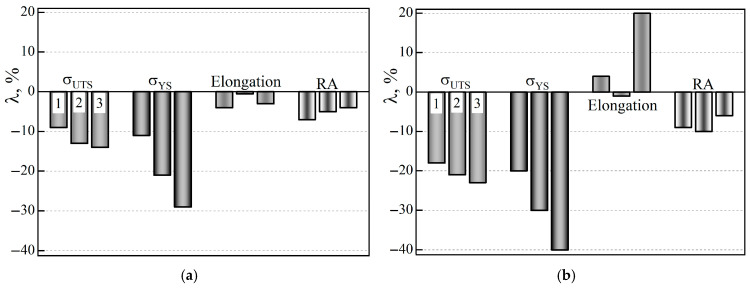
Change in mechanical properties, under tension, of 15CrMoV5-10 steel after approximately 1.9 × 10^5^ h of operation on the main steam pipelines of TPP, relative to its corresponding characteristics in the initial state, determined at different levels along the wall thickness of pipes No. 2 (**a**) and No. 3 (**b**) near their outer (1) and inner (3) surfaces and in the center of the sections (2).

After operation, the steel’s mechanical properties at different levels along the wall thickness of pipes No. 2 and No. 3 changed significantly, relative to the properties at the corresponding levels for steel from non-operated pipe No. 1 (Figure 6). A decrease in the strength characteristics of both variants of operated pipes was detected throughout their entire cross-section, with an obvious increase in the negative effect of steel weakening from their outer to inner surfaces. Consequently, after a practically identical service life, but with a simultaneously smaller (No. 2) or greater (No. 3) number of unit shutdowns, the strength characteristics of the steel decreased maximally in the vicinity of the inner surface of the pipes (σ_UTS_ by 14 and 23%, and σ_YS_ by 29 and 40%, respectively). It was believed that such uneven weakening of both operated steel variants is due to changes in their structure. Overall, in larger grains, the power of dislocation pileups increases, which promotes the propagation of plastic deformation to neighboring grains. However, this cannot explain the more noticeable weakening of steel near the inner surface of the operated pipes relative to the outer one. Therefore, it was assumed that the obtained effect of changing the strength characteristics along the wall thickness of the operated pipes can also be due to more effective embrittlement of steel. Overall, on the surfaces of pipes directly in contact with the process environment, the conditions for hydrogen absorption and, as a consequence, hydrogenation of steel, are more favorable. It is known that the hydrogen content in steel previously operating in steam pipelines is higher than in un-operated steel [39]. In addition, its local concentration in the vicinity of operational fractures of pipe elements exceeds its integral content when it is far from them [40]. And, finally, hydrogen tends to localize in the vicinity of crack-like defects, where its content can significantly exceed the integral content in the metal [51,52]. Indeed, direct measurements of the hydrogen content at different levels along the wall thickness of pipe No. 3 revealed its growth in the direction from the outer to the inner surface. In particular, the hydrogen content reached 1.3 ± 0.1 and 2.2 ± 0.1 ppm in the vicinity of the outer and inner surfaces of pipe No. 3, respectively, whereas in the entire cross-section of pipe No. 1, its integral content, determined at a temperature of 900 °C, did not exceed 1.0 ± 0.1 ppm. In general, such content is consistent with its average content of 0.9–1.8 ppm in steels [53]. However, taking into account the possible localization of hydrogen in the pre-destruction zone at the tips of crack-like defects in the cross-section of operated pipes, its content could increase many times and enhance the negative effect of hydrogen absorbed by steel on its mechanical properties.

It is also known that hydrogenation of steels usually effectively reduces their ductility characteristics [54]. In our study, at all levels of wall thickness of both operated pipes, only a slight decrease in the RA value of steel was recorded, with an almost imperceptible gradient along their thickness (Table 1). In general, the decrease in the RA value for steel in pipe No. 2 was somewhat less than in pipe No. 3. It was believed that this could be a consequence of the combined effect of both a greater number of thermal cycles due to block shutdowns and a higher hydrogen content absorbed by the steel from pipe No. 3. As for the value of elongation, for the steel used in pipe No. 3, instead of a decrease, a small increase was unexpectedly recorded (Figure 6b). Previously, a similar increase in elongation was recorded for metal from a welded joint [38] and a pipe bend [55], which had been in long-term operation on the main steam pipelines of TPP. It was believed that the unconventional increase in elongation of steel from pipe No. 3 (instead of its decrease, which is typical for steel from pipe No. 2) is due to the facilitation of uniform plastic deformation of specimens during their tensile testing. The reason for this effect was the opening of the edges of structural defects that had formed inside the metal at the stage of its operation. Overall, a greater number of unit shutdowns contributes to the formation of such internal defects. Therefore, the identified positive effect of degradation on the elongation value of steel in pipe No. 3 should be considered an illusion rather than an increase in plasticity. Overall, as a rule, both characteristics of plasticity of steels in the initial state either increase or decrease under the influence of such factors as alloying, heat treatment, various hardening technologies, etc. However, it turned out that, due to the degradation of the exploited steel, such a property of both characteristics of plasticity can be violated. In particular, in the case of critically degraded steels (steel from pipe No. 3), elongation no longer characterizes their plasticity. In addition, in the presence of thermal cycles, hydrogen absorbed by the steel contributed to the decohesion of inclusions from the matrix with the formation of defects along the grain boundaries. Considering the gradient of its content along the wall thickness of pipe No. 3, the effect of additional opening of defects in the metal from the vicinity of its inner wall seems quite reliable.

**Figure 7:**
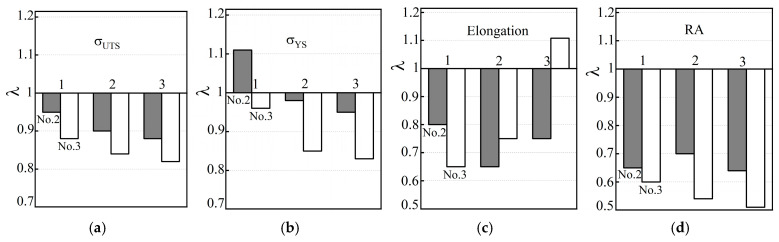
Change in the ultimate strength σ_UTS_ (**a**) and yield strength σ_YS_ (**b**), as well as elongation (**c**) and reduction in area RA (**d**) of pre-hydrogenated (before tensile testing in air) 15CrMoV5-10 steel, which was operating for a long time near the outer (1) and inner (3) surfaces and in the center of the cross-sections (2) of pipes No. 2 (dark) and No. 3 (light columns), relative to the corresponding characteristics of each of these steel variants, obtained by tensile testing in air of non-hydrogenated specimens.

To determine the trend of changes in the mechanical properties of the operated steel variants due to their possible additional hydrogenation from the process environment, the specimens were additionally electrolytically hydrogenated before tensile testing in air (Figure 7). It was noted that after hydrogenation of steel specimens from pipe No. 3, both strength characteristics worsened at all levels of the pipe wall thickness, relative to the corresponding values of non-hydrogenated steel. Only in specimens cut near the outer surface of pipe No. 2 was a slight increase in σ_YS_ of the steel recorded, due to its hydrogenation. In general, a general pattern was noted in the influence of hydrogenation of steels from both operated pipes on their strength characteristics: the negative effect of hydrogenation was more pronounced on the inner than on the outer surface of the pipes (Figure 7a,b). The minor positive effect of hydrogenation of steel used near the outer surface of pipe No. 2 (relative to the non-hydrogenated version of this steel) was considered as a manifestation of hydrogen-induced plasticity in less intensively degraded steel than steel from pipe No. 3 (Figure 7b).

As for the plasticity characteristics of the steel used in pipes No. 2 and No. 3, under the influence of additional hydrogenation of the specimens before testing in air, their elongation and RA values decreased significantly over almost the entire wall thickness of both pipes (Figure 7c,d). In particular, as a result of the hydrogenation of steel specimens from pipe No. 3, their *RA* value decreased, relative to non-hydrogenated steel from 40 to 59% near the outer and inner surfaces of the pipe, respectively. At the same time, their elongation along the pipe wall thickness changed ambiguously (from a decrease of 35% near the outer surface of the pipe to an increase of 11% near its inner surface). It was believed that the increase in the elongation of the steel of pipe No. 3 was due to the large number of operational defects inside it, as favorable places for hydrogen accumulation. Hydrogen could contribute to the additional opening of the edges of these defects during active loading of the specimens, thereby creating the illusion of an increase in their plasticity. Thus, it has been confirmed that additional hydrogenation of steels, which cannot be avoided during their long-term operation on steam pipelines, will contribute to the deterioration of all their properties. And it is especially important that this will occur unevenly across the thickness of the pipe wall. In particular, the negative effect of hydrogen will be most pronounced near the inner surface of the pipes, which is in direct contact with the coolant, which actually ensures a higher hydrogen content here.

**Figure 8:**
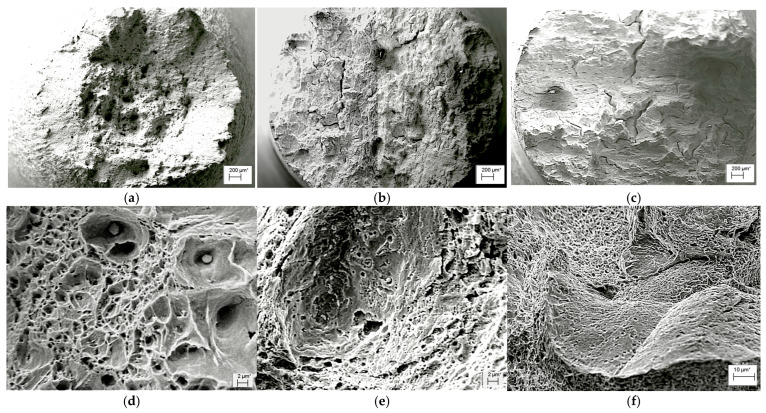
Macro (**a**–**c**) and micro (**d**–**f**) fractograms of 15KhMF5-10 steel specimens cut near the outer surface of pipes No. 1 (**a**,**d**), No. 2 (**b**,**e**) and No. 3 (**c**,**f**) tested for tension in air after preliminary electrolytic hydrogenation.

*Fractographic signs of degradation and hydrogenation of exploited steel.* Fractographic analysis of steel fractures of all three pipe variants (No. 1, No. 2 and No. 3) without preliminary hydrogenation of tensile specimens showed that, in general, a ductile fracture mechanism was realized throughout the entire wall thickness of these pipes. However, after preliminary hydrogenation, only the steel in the initial state retained macro-signs of ductile fracture of the “cup–cone” type, with a ductile relief of dimples at the micro level (Figure 8a,d). Conical sections disappeared from the macro-fractures of steel from both variants of operated pipes, and secondary macro-cracks additionally appeared on them (Figure 8b,c). Microscopic signs of the effect of hydrogen on the fracture of the specimen from pipe No. 2 were oval fragments in the form of large, but at the same time flat, dimples, clearly visible against the background of a generally fine-dimpled relief (Figure 8d). They are interpreted as disc-shaped cracks that grew under the influence of hydrogen accumulated in operational defects in the form of pores formed due to decohesion of large carbides from the matrix. On the fracture of the specimen from pipe No. 3, hydrogenation was manifested by areas with atypical intergranular facets, visible against the background of a ductile dimpled relief (Figure 8d). Traces of carbides were visualized on the surface of these facets, which were precipitated and coagulated at the boundaries of these grains during the operation of the steel. These intergranular facets on the fracture were interpreted as visualized defects of operational origin. The opening of the edges of these defects actually created the illusion of an increase in the elongation of the specimens from pipe No. 3.

*Features of change in fracture toughness of 15CrMoV5-10 steel across the thickness of the pipe wall.* It is known that the characteristics of fracture mechanics (fracture toughness and threshold range of stress intensity factor at the fatigue-crack tip) are more sensitive to operational degradation of the microstructure of heat-resistant steels, compared to their strength and plasticity characteristics in tensile tests [9]. The levels of fracture toughness of the studied variants of 15CrMoV5-10 steel, which, even after operation, was characterized by an elastic–plastic loading diagram, were determined using the *J*-integral method, which allows testing of specimens of relatively small sizes. On both side surfaces of the specimens, mechanical grooves up to 1 mm deep were additionally applied. In this way, favorable conditions were created for quasi-static crack growth in a single section of the specimen. Due to side grooves, the stress–strain state at the tip of the initial fatigue cracks in this section of the specimens was close to the plane-strain state. Thus, it was possible to significantly reduce the effect of the specimen thickness on the fracture toughness of the analyzed steel variants.

**Figure 9:**
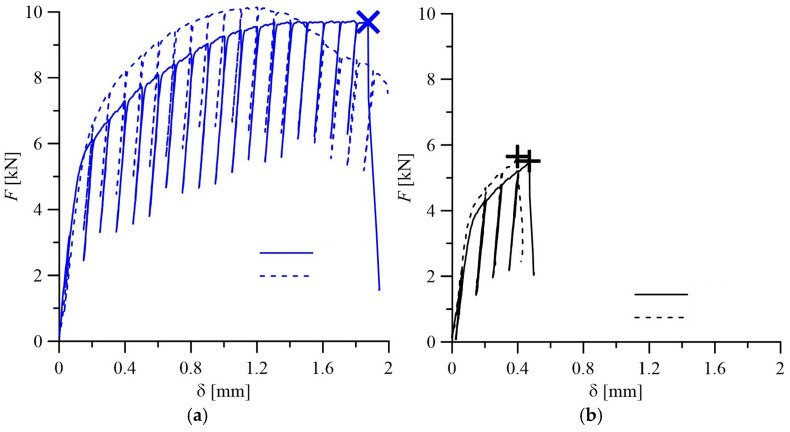
Typical loading diagrams in the coordinates load *F*—displacement at the point of force application δ, recorded during fracture-toughness tests of specimens from the vicinity of the outer (solid line) and inner (dashed line) surfaces of 15CrMoV5-10 steel pipes in the initial state ((**a**) pipe No. 1) and after operation, for approx. 1.9 10^5^ h ((**b**) pipe No. 3).

**Figure 10:**
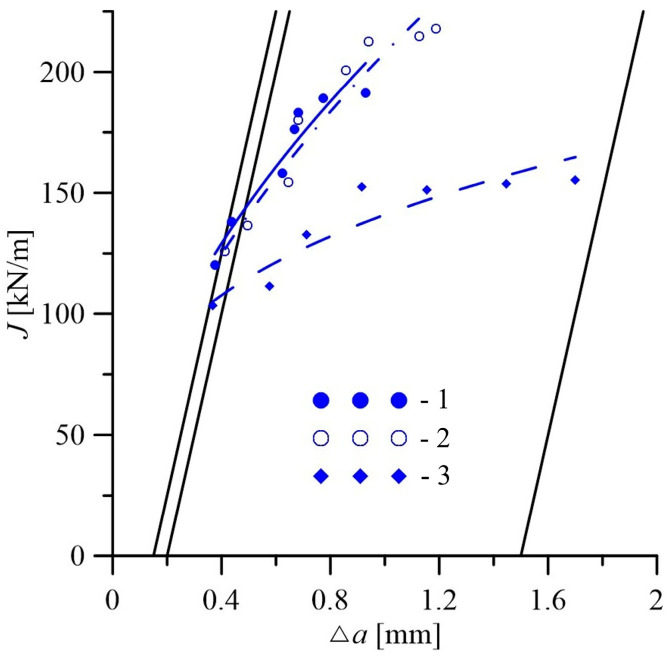
Typical *J*_R_-curves in coordinates for the current values of the *J*-integral *J*_I_ depending on the corresponding crack increments Δ*a*, obtained on tangentially oriented specimens cut near the outer (1) and inner (3) surfaces and in the center of the wall section (2) of pipe No. 1, made of 15CrMoV5-10 steel, in the initial state.

To determine the fracture toughness of 15CrMoV5-10 steel in the initial state (pipe No. 1) and after operation in steam pipelines, loading diagrams of specimens were used, recording the change in their deflection δ at the point of application of load *F* (Figure 9). The *J_R_*-curves (dependencies *J*_I_ = f(Δ*a*), characterizing the current value of the *J*-integral *J*_I_ from the corresponding crack increment Δ*a*), obtained for steel in the initial state (Figure 10), were used to determine the values of *J*_IC_ responsible for the start of quasi-static growth in it. Their values at each of the three levels of determining *J*_IC_ by the thickness of the pipe wall are given in Table 1. In the exploited steel, their destruction occurred with a slight deviation of the loading diagrams of the specimens from linearity (Figure 9b). It is clear that, in this case, it was difficult to apply the method of partial unloading of the specimens to construct the corresponding *J*_R_-curves. Therefore, the fracture toughness of exploited steel was determined by the energy determined at the moment of specimen fracture as *J*_IC_ = 2*E*/*B_e_* × *b* (*E* is the energy expended on specimen fracture and determined from their loading diagrams; *B_e_* = (*B* × *B_n_*)^1/2^ is the effective thickness of notched specimen, where *B* and *B_n_* are, respectively, the nominal thickness of the specimen and its actual thickness, taking into account the presence of side grooves on its surfaces; *b* = *W* − *a*; and *W* is the height of the specimen. The fracture-toughness values of steel determined in this way at three levels along the wall thickness of both operated pipes are also given in Table 1.

**Figure 11:**
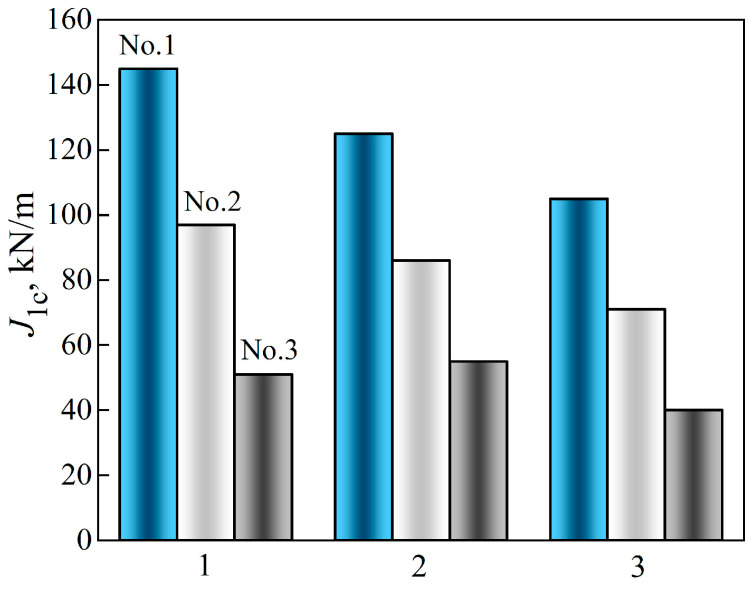
Values of fracture toughness *J*_IC_ of 15CrMoV5-10 steel determined on tangentially oriented specimens cut in the vicinity of the outer (1), and inner (3) surfaces, and in the center of the cross-sections (2) of pipes made of 15CrMoV5-10 steel in the initial state (No. 1) and after 1.9 × 10^5^ h of operation on steam pipelines with 502 (No. 2) and 576 (No. 3) shutdowns.

The analysis of the obtained results showed the presence of a fracture-toughness gradient of the analyzed steel across the wall thickness of the studied pipe variants. A fairly significant difference in the *J*_IC_ value in the direction from the outer to the inner surface of the pipe was observed, even for 15CrMoV5-10 steel in the initial state, from pipe No. 1 (Figure 11). As a consequence, the *J*_IC_ value of this steel, determined on specimens from the vicinity of the inner surface of the pipe, was 28% lower than from the vicinity of its outer surface. The revealed tendency to decrease the fracture-toughness values of steel in the initial state across the pipe wall thickness is consistent with the observed tendency towards a slight decrease in its plasticity characteristics.

In this case, the *J*_IC_ values of the operated steel (pipes No. 2 and No. 3) at all levels of pipe wall thickness were lower than those of the steel in the initial state (Figure 11). This is direct evidence of a change in the critical stress–strain state at the crack tip depending on the structural state of the steel, caused by its degradation under the influence of operational factors. Moreover, the discrepancy between the operating conditions of the steel near the outer and inner surfaces of the pipes had a different effect, including on its ability to resist fracture. Indeed, the outer surface of the pipe contacts the air, increasing surface oxidation and decarburization of steel [56], whereas active hydrogenation of metal is more easily carried out from the inner surface of the pipes, which are in contact with high-parameter steam [40]. Hydrogen absorbed by the metal intensifies the diffusion redistribution of both carbon and alloying elements [31]. Therefore, it was considered a factor contributing to the degradation of the steel structure, with the precipitation and coagulation of carbides at the grain boundaries and the weakening of ferrite. Thus, although both analyzed factors have a detrimental effect on steel, contributing to its weakening, due to the significant difference in the diffusion coefficients of oxygen and hydrogen, the effectiveness of their impact is not the same.

It was also noted that in both variants of the operated pipes, the tendency to decrease fracture toughness in the direction from the outer to the inner surface was preserved. However, the *J*_IC_ values of steel operated with a smaller number of shutdowns (pipe No. 2) decreased to almost the same extent (by 31–33%) at all levels of pipe wall thickness analysis, relative to the corresponding *J*_IC_ values of the steel in the initial state (Figure 11). Meanwhile, the fracture toughness of steel operated in pipe No. 3, with a greater number of process shutdowns, decreased significantly more. Thus, the maximum decrease in the *J*_IC_ value relative to the corresponding value for the steel of pipe No. 1 occurred near the outer surface of pipe No. 3 (by 65%). At the same time, in the center of the pipe wall, the decrease in fracture toughness was the smallest (by 56%), and near the inner surface it reached 62%. In this case, the decrease in the *J*_IC_ values of steel from pipe No. 3, caused by a large number of unit shutdowns compared to pipe No. 2, reached 47, 43 and 55%, respectively, near the outer and inner surfaces and in the center of their walls. Since the service life of steel in both pipes was almost the same, this effect was considered a direct consequence of the increase in the number of unit shutdowns during the operation of steel in pipe No. 3. Thus, firstly, the reduction in the fracture toughness of steel during operation due to the increase in the number of such shutdowns was evident across the entire cross-section of the pipe wall, and secondly, this confirmed the intensification of steel degradation under their influence near the outer surface of the pipes. Overall, such a significant decrease in the fracture toughness of 15CrMoV5-10 steel due to its degradation threatened the unpredictable destruction of the steam pipeline, and thus confirmed the timeliness of decommissioning pipeline No. 3.

An important consequence of the operational degradation of steel was also the gradual decrease in the fracture-toughness gradient across the thickness of the pipe wall (Figure 11). Overall, in the initial state, there was an obvious gradient of *J*_IC_ values in the direction from the outer to the inner surface of the pipe. After the operation with a smaller number of unit shutdowns, it decreased somewhat, but was still obvious. After a larger number of them, the fracture toughness of 15CrMoV5-10 steel remained practically the same across almost the entire thickness of the pipe wall. It was believed that a decrease in the *J*_IC_ value by almost three times (and across practically the entire wall thickness of pipe No. 3) is a sign of exhaustion of the steel resource, due to the complex influence of operational factors.

**Figure 12:**
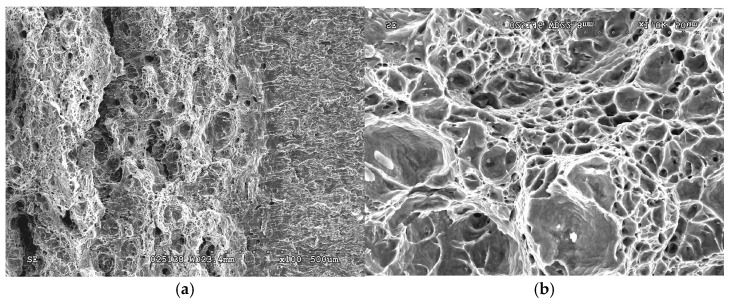
Typical macro (**a**) and micro (**b**) fractograms of a fracture of a 15CrMoV5-10 steel in the initial state (No. 1), tested for fracture toughness. Quasi-static crack growth on the fractograms occurred from right to left.

*Fractographic features of specimen fractures after fracture-toughness tests.* Regardless of the specimen arrangement along the wall thickness of pipe No. 1, a characteristic ductile relief of quasi-static crack growth was observed on their fractures, following the stretched zones at the tips of fatigue cracks. Such relief was formed by the mechanism of nucleation, growth, and merging of the nearest micro voids by rupture of the bridges between them (Figure 12).

**Figure 13:**
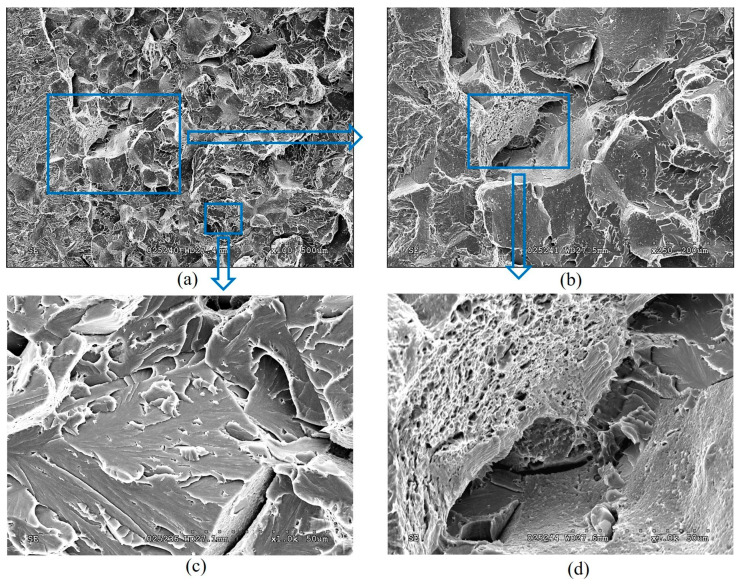
Typical macro (**a**) and micro (**b**–**d**) fractograms at different magnifications of a 15CrMoV5-10 steel specimen after 1.9 × 10^5^ h of operation on steam pipelines of a TPP with a smaller number of unit shutdowns (No. 2), tested for fracture toughness. Quasi-static crack growth on the fractograms occurred from left to right.

**Figure 14:**
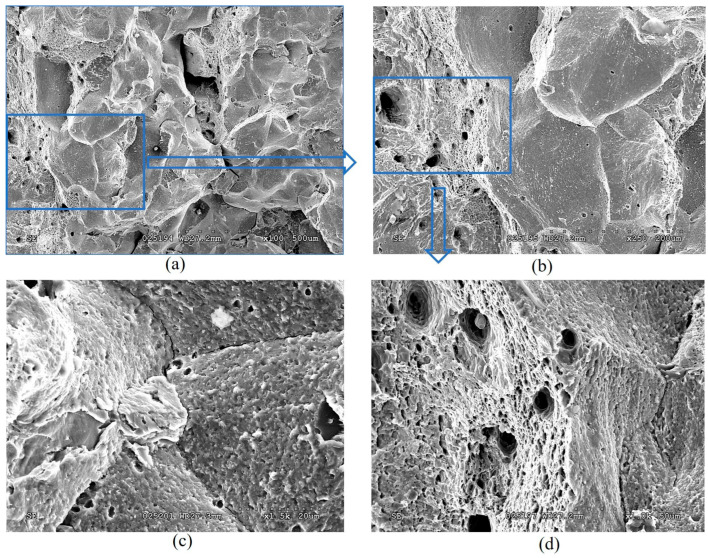
Typical macro (**a**) and micro (**b**–**d**) fractograms at different magnifications of a 15CrMoV5-10 steel specimen after 1.9 × 10^5^ h of operation on steam pipelines of a TPP with a smaller number of unit shutdowns (No. 3), tested for fracture toughness. Quasi-static crack growth on the fractograms occurred from left to right.

The stretched zones at the tips of the initial fatigue cracks on the fractures of steel operated in pipes No. 2 and No. 3 were practically not observed, whereas areas of quasi-static crack growth due to the ductile mechanism were observed along their entire front, but only in the form of short sections of dimple fracture (Figure 13a and Figure 14a). Only a slightly larger number of similar local areas of crack growth by the dimple mechanism were observed on the specimens from pipe No. 2 (after fewer shutdowns during operation) compared to pipe No. 3. A similar, although less clearly expressed, tendency to a decrease in the number of areas of ductile crack growth was noted in the direction from the outer to the inner surface of the operated pipes, which is consistent with the results of mechanical tests for fracture toughness (Figure 11).

Significant differences in the fracture mechanisms of the specimens from both operated pipes were revealed only at the stage of spontaneous crack propagation in them. Thus, crack propagation over the entire cross-section of specimens from pipe No. 2 (with a smaller number of process shutdowns) occurred by a mixed intergranular and transgranular mechanism (Figure 13), whereas in specimens from pipe No. 3 (with a larger number of shutdowns), the intergranular fracture mechanism became practically dominant over the entire fractures (Figure 14). Consequently, the brittle fracture mechanism (intergranular or transgranular) prevailed in the fractures of specimens from both variants of operated pipes after their fracture-toughness tests. It was considered that the dominance of intergranular fracture in steel fractures after a large number of shutdowns indicated a loss of cohesive strength of adjacent grains over a significant part of the cross-section of specimens from pipe No. 3. Meanwhile, in specimens from pipe No. 2, the proportion of intergranular fracture was significantly smaller, and, therefore, in this variant of steel, total weakening of the bonds between adjacent grains had not yet occurred.

Thus, it is fractographically confirmed that there is a different energy intensity of fracture of specimens made of 15CrMoV5-10 steel after its operation on steam pipelines of TPP with different numbers of process shutdowns. At the initial stage of quasi-static crack growth, the decrease in the energy intensity of fracture of specimens from pipe No. 3 was manifested by a smaller area of ductile crack-growth segments by the dimple mechanism and a greater number of breaks in the front of such growth across the thickness of the specimens, relative to that observed in specimens from pipe No. 2. However, at the stage of fracture propagation over the entire cross-section, a stronger loss of adhesion between adjacent grains was manifested in specimens from pipe No. 3 than in specimens from pipe No. 2 (after a greater and lesser number of block shutdowns, respectively).

It is also important to note that, after a greater number of unit shutdowns during long-term operation of 15CrMoV5-10 steel, signs of degradation cover almost the entire cross-section of the pipe wall. This significantly weakened the cohesion between adjacent grains and contributed to intergranular fracture of the specimens, even at the stage of their spontaneous failure during fracture-toughness tests. Of course, this does not prove that intergranular cracking covered almost the entire cross-section of the pipe. But there is no doubt that weakening of the grain boundaries did occur. Moreover, with a smaller number of unit shutdowns during the same service life of steel, the number of intergranular fragments at the fracture of the specimens was significantly smaller. Thus, the study of changes in properties across the wall thickness of steam pipeline pipes and the determining fracture mechanisms associated with steel weakening made it possible to study in more detail the nature of the fracture of degraded steels.

## 4. Conclusions

Regularities of changes in structural characteristics (size of grains and carbides on their boundaries) along the thickness of steam pipelines of TPP in the initial state and after long-term operation with different numbers of unit shutdowns have been established. The unevenness of the change in structural characteristics across the pipe cross-section, and the inverse pattern of change in steel hardness relative to them, are shown.

A gradient of strength, plasticity and fracture-toughness characteristics was found across the wall thickness of pipes made of 15CrMoV5-10 steel, both in the initial state and after its long-term operation (1.9 × 10^5^ h) on steam pipelines of TPP. The effect of steel weakening due to its degradation was most clearly manifested on the inner surface of the operated pipes. Both steel ductility characteristics decreased symbiotically, after a smaller number of block shutdowns. However, after a larger number of shutdowns, the *RA* characteristic decreased even more, while the elongation near the inner surface of the pipe increased by 20%, relative to unexploited steel. This was explained by the opening of the edges of internal defects that arose during the operation of the steel on the steam pipeline, which created the illusion of increased plasticity of the steel during tensile testing of specimens from pipe No. 3. Preliminary electrolytic hydrogenation of the specimens before tensile testing in air demonstrated even more clearly the gradient of all mechanical properties across the thickness of the pipe wall.

A significant decrease in the fracture toughness *J*_IC_ of 15CrMoV5-10 steel was found, caused by the intensification of steel degradation under the impact of unit shutdowns during long-term operation on the main steam pipelines of a TPP. Despite the same service life of steel on steam pipelines (1.9 × 10^5^ h), the maximum decrease in the *J*_IC_ level on tangentially oriented specimens was recorded near the outer and inner surfaces of the pipe (by almost 65 and 61%), which was operated with a greater number of shutdowns compared to the characteristics of steel in the initial state. With a smaller number of shutdowns, the decrease in *J*_IC_ reached 32–33% near both surfaces of the pipe.

The disruption of the continuity of the front and the decrease in the depth of quasi-static crack growth behind the ductile dimpled mechanism, as well as the change of transgranular fracture to intergranular one, at the stage of spontaneous propagation of fracture over the entire cross-section of specimens tested for fracture toughness, were considered fractographic signs of intensification of steel degradation with an increase in the number of block shutdowns during its operation. The dominance of the intergranular fracture mechanism in steel operated with a greater number of block shutdowns indicated a decrease in the cohesive strength between adjacent grains, which covered almost the entire cross-section of the pipe. This was considered a sign of reaching a critical state of steel degradation.

## Figures and Tables

**Table 1:** Mechanical properties of the analyzed steel 15CrMoV5-10 of TPP steam pipelines.

Pipe Designation	Level of Specimen Location in Cross-Section of Pipe (According to Figure 1)	σ_UTS_, MPa	σ_YS_, MPa	Elongation, %	*RA*, %	*J*_IC_, kN/m
No. 1	1	584	306	29.3	77.5	145
	2	603	326	26.7	76.1	125
	3	592	341	26.1	76.3	105
No. 2	1	531	272	28.1	72.2	97
	2	525	259	26.6	72.4	86
	3	512	242	26.9	73.1	71
No. 3	1	476	244	30.5	70.8	51
	2	474	228	26.5	68.6	55
	3	457	203	31.2	71.7	40

## Data Availability

The original contributions presented in this study are included in the article. Further inquiries can be directed to the corresponding author.

## Abbreviations

The following abbreviations are used in this manuscript:

TPP	Thermal power plants
SEM	Scanning electron microscope
*D*	Grain size
*d*	Carbide size
*HB*	Hardness
*RA*	Reduction in area
σ_UTS_	Ultimate strength
σ_YS_	Yield strength
*El*	Elongation
*t*	Pipe wall thickness

## References

[1] Dmytrakh I.M., Syrotyuk A., Hembara O., Hrynenko M.V. (2024). Effect of hydrogen concentration in metal on residual durability of defected pipelines. Procedia Struct. Integr..

[2] Andreykiv O., Hembara O., Dolinska I., Sapuzhak Y., Yadzhak N., Bolzon G., Gabetta G., Nykyforchyn H. (2021). Prediction of residual service life of oil pipeline under non-stationary oil flow taking into account steel degradation. Degradation Assessment and Failure Prevention of Pipeline Systems.

[3] Zieliński A., Golański G., Sroka M. (2017). Influence of long-term ageing on the microstructure and mechanical properties of T24 steel. Mater. Sci. Eng. A.

[4] Dobrzanski J., Hernas A., Moskal G., Viswanathan R., Bakker W., Cole R. (2011). Microstructural degradation in boiler steels: Materials developments, properties and assessment. Power Plant Life Management and Performance Improvement.

[5] Výrostková A., Kepič J., Macko R., Homolová V. (2011). Degradation of 0.5Cr-0.5Mo-0.25V steel microstructure during exploitation. Mater. Eng.–Materiálové Inžinierstvo.

[6] Krechkovs’ka G.V. (2008). Structural changes in the exploitation of steam power plant pipeline 15Kh1M1F-type steel concerning with shut downs of power units. Metallofiz. Noveish. Tekhnol..

[7] Krechkovska H., Student O., Zvirko O., Hredil M., Svirska L., Tsybailo I., Solovei P. (2023). Substantiation of the critical structural and mechanical state of low-alloy heat-resistant steel from steam pipelines of thermal power plant. Eng. Fail. Anal..

[8] Dzioba I. (2010). Properties of 13KhMF steel after operation and degradation under laboratory conditions. Mater. Sci..

[9] Romaniv O.M., Nykyforchyn H.M., Dzioba I.R., Student O.Z., Lonyuk B.P. (1998). Effect of damage in service of 12Kh1MF steam-pipe steel on its crack resistance characteristics. Mater. Sci..

[10] Maruschak P.O., Baran D.Y., Sorochak A.P., Bishchak R.T., Yasnii V.P. (2012). Cyclic crack resistance and micromechanisms of fracture of steel 25Kh1M1F. Strength Mater..

[11] Neimitz A., Galkiewicz J., Dzioba I. (2010). The ductile to cleavage transition in ferritic Cr-Mo-V steel: A detailed microscopic and numerical analysis. Eng. Fract. Mech..

[12] Dzioba I., Gajewski M., Neimitz A. (2010). Studies of fracture processes in Cr-Mo-V ferritic steel with various types of microstructures. Int. J. Press. Vessels Pip..

[13] Liu J., Li Y. (2022). Influence of 12Cr1MoV material on tissue properties at high temperature and long operating time. Processes.

[14] Jasiński A., Zieliński A., Purzyńska H. (2018). Residual life of repair welded joints in pipelines made of 13HMF after use for the design operating time. Prace Inst. Met. Żelaza.

[15] Dobrzanski J., Zielinski A., Krtzon H. (2007). Mechanical properties and structure of the Cr-Mo-V low-alloyed steel after long-term service in creep condition. J. Achiev. Mater. Manuf. Eng..

[16] Student O.Z., Krechkovs’ka H.V., Palashchuk T.E., Hladkyi Y.M. (2018). Influence of the long-term operation of 12Kh1MF steel of the bends of main steam pipelines of thermal power plants on its mechanical properties. Mater. Sci..

[17] Yasniy O., Vuherer T., Yasniy V., Sobchak A. (2013). Mechanical behaviour of material of thermal power plant steam superheater collector after exploitation. Eng. Fail. Anal..

[18] Hu Z.-F., Rasul M. (2012). Part 10. Heat-resistant steels, microstructure evolution and life assessment in power plants. Thermal Power Plants.

[19] Smiyan O.D., Student O.Z. (2021). Fractographic signs of gigacycle fatigue and hydrogenation of heat-resistant steels after long-term operation. Mater. Sci..

[20] Nykyforchyn H.M., Student O.Z., Krechkovs’ka H.V., Markov A.D. (2010). Evaluation of the influence of shutdowns of a technological process on changes in the in-service state of the metal of main steam pipelines of thermal power plants. Mater. Sci..

[21] Hidalgo J., Vittorietti M., Farahani H., Vercruysse F., Petrov R., Sietsma J. (2020). Influence of M23C6 carbides on the heterogeneous strain development in annealed 420 stainless steel. Acta Mater..

[22] Ney José Luiggi A. (2019). Study of elastic, structural, electronic, magnetic, and topological properties of η-Fe_2_C carbide under pressure. J. Phys. Chem. Solids.

[23] Claesson E., Magnusson H., Kohlbrecher J., Thuvander M., Hedström P. (2022). Evolution of iron carbides during tempering of low-alloy tool steel studied with polarized small angle neutron scattering, electron microscopy and atom probe. Mater. Charact..

[24] Fang C.M., Sluiter M.H.F., van Huis M.A., Ande C.K., Zandbergen H.W. (2010). Origin of predominance of cementite among iron carbides in steel at elevated temperature. Phys. Rev. Lett..

[25] Kobayashi S., Fukunishi H. (2020). Reduction of thermal expansion of ferritic/martensitic heat resistant steels—Alloying effects on thermal expansion of α-Fe phase. ISIJ Int..

[26] Duan J., Hou T., Zhang D., Wu K. (2023). Insights to the fracture toughness, damage tolerance, electronic structure, and magnetic properties of carbides M_2_C (M = Fe, Cr). Mater. Res. Express.

[27] Baltušnikas A., Levinskas R., Lukošiūtė I. (2008). Analysis of heat resistant steel state by changes of lattices parameters of carbides phases. Mater. Sci. (Medžiagotyra).

[28] Krechkovs’ka H.V., Student O.Z., Nykyforchyn H.M. (2019). Diagnostics of the engineering state of steam pipelines of thermal power plants by the hardness and crack resistance of steel. Mater. Sci..

[29] Taler J., Zima W., Jaremkiewicz M. (2016). Simple method for monitoring transient thermal stresses in pipelines. J. Therm. Stresses.

[30] Taler J., Węglowski B., Pilarczyk M. (2017). Monitoring of thermal stresses in pressure components using inverse heat conduction methods. Int. J. Numer. Methods Heat Fluid Flow.

[31] Fedorov V.V. (2010). Influence of hydrogen on the phase composition and physicomechanical properties of structural materials. Mater. Sci..

[32] Vorobel R., Student O., Ivasenko I., Maruschak P., Krechkovska H., Zvirko O., Berehulyak O., Mandziy T., Tsybailo I., Solovei P. (2024). Development of a method for computer processing of fractographic images to assess the cohesion of inclusions to the matrix in the weld metal after its operational degradation and hydrogenation. Materialia.

[33] Babii L.O., Student O.Z., Zagorski A., Markov A.D. (2007). Creep of degraded 2.25 Cr-Mo steel in hydrogen. Mater. Sci..

[34] Marushchak P.O., Bishchak R.T., Hlykha V., Sorochak A.P. (2011). Influence of temperature on the impact toughness and dynamic crack resistance of 25Kh1M1F steel. Mater. Sci..

[35] Balyts’kyi O.I., Ripei I.V., Onyshchak O.Y. (2009). Variations of the impact toughness of 12Kh1MF steel in operating steam pipelines of thermal power plants. Mater. Sci..

[36] Ostash O.P., Vol’demarov O.V., Hladysh P.V., Ivasyshyn A.D. (2010). Evaluation of the degradation of steels of steam pipelines according to their structural, mechanical, and electrochemical characteristics. Mater. Sci..

[37] Romaniv O.N., Tkach A.N., Dzioba I.R., Simin’kovich V.N., Islamov A.A. (1989). Effect of long-term thermomechanical treatment on the crack resistance of 12Kh1MF steel. Mater Sci.

[38] Krechkovs’ka H.V. (2016). Fractographic signs of the mechanisms of transportation of hydrogen in structural steels. Mater. Sci..

[39] Hutsaylyuk V., Student O., Maruschak P., Krechkovska H., Zvirko O., Svirska L., Tsybailo I. (2023). Analysis of mechanical properties of welded joint metal from TPP steam piping after its operational degradation and hydrogenation. Materials.

[40] Melekhov R.K., Vasylyk A.V., Palashchuk E.I., Krutsan H.M., Onyshchak Y.D. (2004). Some specific features of degradation of superheater tubes of the boiler of the thermal electric power plants made of 12Kh18N12T. Mater. Sci..

[41] (2013). Seamless Steel Tubes for Pressure Purposes. Technical Delivery Conditions. Part 2. Non-Alloy and Alloy Steel Tubes with Specified Elevated Temperature Properties.

[42] (2020). Metallic Materials—Tensile Testing—Part 1: Method of Test at Room Temperature.

[43] (2018). Metallic Materials—Calibration and Verification of Static Uniaxial Testing Machines—Part 1: Tension/Compression Testing Machines—Calibration and Verification of the Force-Measuring System.

[44] (2012). Metallic Materials—Calibration of Extensometer Systems Used in Uniaxial Testing.

[45] (1996). Standard Test Method for J-Integral Characterization of Fracture Toughness.

[46] (2011). Standard Test Method for Measurement of Fracture Toughness.

[47] (2015). Standard Test Method for Measurement of Fracture Toughness.

[48] Miyata K., Sawaragi Y. (2001). Effect of Mo and W on the phase stability of precipitates in low Cr heat resistant steels. ISIJ Int..

[49] Yamada K., Igarashi M., Muneki S., Abe F. (2003). Effect of Co addition on microstructure in high Cr ferritic steels. ISIJ Int..

[50] Baltušnikas A., Levinskas R., Lukoštūtė I. (2007). Kinetics of carbide formation during ageing of pearlitic 12X1MF steel. Mater. Sci. (Medžiagotyra).

[51] Dmytrakh I.M., Smiyan O.D., Syrotyuk A.M., Bilyy O.L. (2013). Relationship between fatigue crack growth behaviour and local hydrogen concentration near crack tip in pipeline steel. Int. J. Fatigue.

[52] Hembara O., Syrotyuk A., Chepil O., Sapuzhak Y., Hembara N. (2024). Evaluation of increased local hydrogen concentration in the vicinity of various types of defects in low-alloyed steels. Procedia Struct. Integr..

[53] Birnbaum H.K. (1997). An overview of hydrogen failure mechanisms. Nav. Res..

[54] Liu J., Zhao M., Rong L. (2023). Overview of hydrogen-resistant alloys for high-pressure hydrogen environment: On the hydrogen energy structural materials. Clean Energy.

[55] Tsybailo I.O., Svirska L.M., Solovei P.R., Krechkovska S.R., Datsko B.M., Student O.Z. (2023). Use of electrolytic hydrogenation to visualize the damage of long-term operated heat-resistant steel of TPP steam pipelines. Mater. Sci..

[56] Ostash O.P., Kondyr A.I., Vol’demarov O.V., Hladysh P.V., Kurechko M.V. (2009). Structural microdamageability of steels of the steam pipelines of thermal power plants. Mater. Sci..

